# Targeting JNK to prevent antiretroviral neuropathy

**DOI:** 10.18632/oncotarget.15091

**Published:** 2017-02-04

**Authors:** Nicoletta Galeotti

**Affiliations:** Laboratory of Neuro-psychopharmacology, Department of Neurosciences, Psychology, Drug Research and Child Health, Section of Pharmacology and Toxicology, University of Florence, Florence, Italy

**Keywords:** antiretroviral, neuropathic pain, JNK, BDNF, NRTI, Neuroscience

AIDS, a disease previously associated with rapid death, has become a chronic illness in the developed world. Nucleoside reverse transcriptase inhibitors (NRTIs) in combinations with other antiretrovirals (highly active antiretroviral therapy, HAART) are the cornerstones of AIDS therapy, turning HIV infection into a manageable clinical entity. Life expectancy with HIV in well-resourced countries is now estimated to be up to two-thirds that of the general population [1].

Despite the incidence of most neurological complications of HIV has fallen with the introduction of effective therapy, therapeutic experience revealed serious side effects. Rates of sensory neuropathy (SN) have been rising since the first effective antiretroviral drugs were developed [2], and the associated pain has a major impact on quality of life. HIV-SN is comprised of at least two clinically indistinguishable, and often coexisting, neuropathies: a distal sensory polyneuropathy associated with HIV disease itself, and a distal sensory polyneuropathy associated with antiretroviral treatment, mainly with potentially neurotoxic NRTIs [3]. Recent studies suggest symptomatic SN prevalence rates of 20% to 57% among ambulatory patients with HIV and the prevalence of pain has been reported as high as 50% to 90% [4]. Despite this high rate of pain, treatment of painful HIV-SN is often poor since analgesics used in other forms of neuropathic pain have proven ineffective [5]. Adding to the complexity, terminating NRTI therapy does not always reverse neuropathy and patients continue to suffer from pain, leading the antiretroviral neuropathic pain to become a major impact on quality of life in otherwise largely healthy individuals. With the high global HIV prevalence, and with infected individuals gaining increased access to HAART, painful HIV-SN represents a large and expanding world health problem.

With this is mind, Sanna et al. [6] investigated the signalling pathways involved in the onset and development of the antiretroviral pain hypersensitivity to identify effective pain management strategies to limit discontinuation or abandon of antiretroviral therapy. Increases in BDNF concentrations in the dorsal horn of mice exposed to NRTI represent one of the early events, detected since a few hours after treatment, and this BDNF rise was correlated with pain hypersensitivity and allodynia [6]. However, BDNF influences different neuronal functions. After peripheral nerve damage it contributes to the pathogenesis of neuropathic pain and also activates a regenerative response to the axonal damage [7]. This dual effect makes targeting neurotrophin for the relief of neuropathic pain an approach that may have important safety limitations. Clinical studies of anti-BDNF antibodies for the relief of neuropathic pain conditions are lacking. However, unfavourable side effects emerged during the early phase clinical trials of anti-NGF antibodies and, due to these safety concerns, the United States Food and Drug Administration (FDA) placed all ongoing clinical trials on clinical hold.

Elucidation of the early events in the signalling pathways downstream BNDF would allow to identify a more suitable therapeutic target to counteract the pronociceptive pathway leaving the regenerative response unaltered, thus reducing the incidence of untoward side effects. Increasing body of evidence suggests that the c-Jun N-terminal kinase (JNK) cascade is a critical signalling pathway for the onset and the maintenance of neuropathic pain. Sanna et al. [6] reported a robust overphosphorylation of both JNK1 and JNK2 isoforms since day 1 post antiretroviral injection in the spinal cord dorsal horn, along with a JNK-dependent increase in the phosphorylation of c-Jun, a transcription factor substrate of JNK involved in pain perception. In addition to c-Jun, identified transcription factors implicated in the sensory neuron response to axonal injury include activating transcription factor 3 (ATF3), widely known as marker of neurodegeneration. Co-immunoprecipitation results indicated that ATF3 and c-Jun can act together to regulate transcription through heterodimerization and that their coincident upregulation may act synergistically to induce neuropathic pain.

Sanna et al. [6] showed that treatment with a JNK blocker prevented antiretroviral hyperalgesia by restoring the baseline or normal pain threshold of the neuropathic mice. Particularly relevant is that JNK inhibition prevents the onset and development of neuropathic pain since effective only when occurred before exposition to the neurotoxic antiretroviral. When the neuropathic pain is already established, the JNK blocker was ineffective. A JNK pathway blockade, instead of diminishing the stimulus-dependent response, would prevent that response and a JNK blocker could thus be used as prophylactic therapy to be administered before beginning the antiretroviral therapy to prevent the development of painful SN.

With an estimated 33 million people living with HIV and more gaining access to antiretroviral therapy every day, the management of antiretroviral associated neuropathic pain is a problem of major global significance. Despite the decreased use in developed countries due to neurotoxicity, NRTIs remain in the first-line HIV treatment in many resource-limited countries as a result of its high effectiveness and inexpensiveness. Nevertheless, NRTI-induced neuropathy may precipitate abbreviation or discontinuation of antiretroviral therapy thus limiting viral suppression strategies. Authors [6] indicate JNK as a target to be exploited as a therapeutic approach to intervene and better manage the antiretroviral-induced neuropathic pain. The efficacy of JNK inhibitors to prevent the development of neuropathic pain might represent a unique preventive therapy that might enlarge and prolong the clinical use of these highly effective antiviral drugs, otherwise destined to be phased it out. JNK blockers are currently tested for clinical use for the treatment of several pathologies with a good tolerability profile and may represent a concrete therapeutic opportunity in humans.
